# Frail parents and adult children’s life-satisfaction: a longitudinal analysis of Norwegian data

**DOI:** 10.1007/s10433-025-00883-9

**Published:** 2025-09-29

**Authors:** Morten Blekesaune, Vegard Skirbekk

**Affiliations:** 1 Department of Sociology and Social Work, University of Agder, Post Box 422, NO-4604 Kristiansand, Norway; 2 Centre for Fertility and Health, Norwegian Institute of Public Health, Oslo, Norway; 3 Department of Psychology, University of Oslo, Oslo, Norway

**Keywords:** Frail parents, Caregiving, Life satisfaction, Panel data, Norway

## Abstract

Earlier research has found that adult children’s caregiving for older parents is associated with a decline in life satisfaction. However, other research indicates that emotional stress in adult children might be related to the declining health and frailty of their older parents rather than caregiving per se. Hence, there is a possibility that the first set of findings (declining life satisfaction when giving care) reflects factors not specified in statistical models rather than the care provided by adult children. This study tests this possibility by investigating changes in life satisfaction among 3,094 adult children from panel data in Norway that includes multiple indicators of health and care needs in older parents, together with data on who is providing care. Declining life satisfaction was observed among daughters but not among sons, and these changes were driven by the frailty and care needs of their parents rather than caregiving per se. The findings indicate that it is not caregiving that affects life satisfaction but the circumstances leading to caregiving. In these situations, adult daughters may struggle with sources of distress beyond providing support and care. Further research should investigate these relationships in countries with different distributions of care between families and public care institutions.

## Introduction

Many adult children caring for old and frail parents report low levels of subjective well-being (Pinquart and Sörensen 2004; 2006). Understanding the sources of these problems is important for supporting these adult children. A popular idea is that caregiving as such gives rise to low well-being (Pearlin et al. 1997; Pinquart and Sörensen 2004). Estimating such relationships is complicated, however, as caregiving and co-residence may not be independent variables. Some studies have tried to overcome these issues by using instrumental variables (Kalenkoski and Oumtrakool 2017) or longitudinal data (Hajek and König 2016; Van den Berg et al. 2014). Findings from these studies indicate that caregiving can negatively affect happiness (Kalenkoski and Oumtrakool 2017), perhaps particularly in women (Hajek and König 2016), and it may also negatively affect life satisfaction (Van den Berg et al. 2014).

One limitation of these studies is that they do not distinguish between the impact of having a parent in need of care—which, in some cases, means that they are in a slow and painful process of dying—and the actual provision of care for older parents. This limitation has been addressed by research investigating how the declining health of older parents may also affect the health of their adult children. For example, Amirkhanyan and Wolf (2003) argued that the caring needs of older parents and caregiving activities undertaken by their adult children should be seen as separate sources of stress. Research using longitudinal data on mental health and depressive symptoms has supported this argument (Amirkhanyan and Wolf 2006; Bom et al. 2019; Han 2023). Similar findings have been made regarding loneliness among adult daughters (Van den Broek and Grundy (2018). Some of these studies also indicate that the negative outcomes associated with the declining health and care needs of older parents could be more pronounced in adult daughters than in sons (Amirkhanyan and Wolf 2006; Bom et al. 2019).

Hence, declining well-being (e.g., life satisfaction) when older parents need care can be driven by two sets of situational factors that often overlap. The first relates to the fact that older parents experience declining health, become dependent on help and support from others, and undergo a slow process leading to their death. The second set of factors pertains to the help and support provided by their adult children (Amirkhanyan and Wolf 2003; Wolf et al. 2015; Han 2023).

Our empirical analysis investigates how various indicators of the health and caregiving needs of older parents affect the life satisfaction of their adult children, the cognitive component of subjective well-being (Diener et al. 1999). It expands on previous research on this topic, which has largely investigated mental distress and affective well-being (depression). The cognitive component is more stable and less reactive to daily fluctuations than stress and affective components of well-being (Hudson et al. 2022). This could matter because responsibilities for older parents may not only cause stress but may also affect how adult children balance various responsibilities in their lives (e.g., career, family dynamics).

We use two waves of panel data from Norway, collected 10 years apart (in 2007–08 and 2017–18), and fixed effects regression models. The first hypothesis (H1) is that life satisfaction decreases when parents need care. A second hypothesis (H2) is that life satisfaction decreases when providing care to older parents. A third hypothesis (H3) is that both effects (H1, H2) could be stronger in daughters than in sons. This analysis contributes to previous research primarily by investigating how the health and caregiving needs of older parents affect human well-being in adult children—measured by life satisfaction—additional to morbidity (particularly depressive symptoms), which has been investigated in previous studies.

### Stress and family relations

Much research on caregiving and subjective well-being is based on the stress process model (Broese van Groenou et al. 2013; Zarit and Whitlatch 2023). This model distinguishes between primary stressors—arising from the responsibilities and tasks associated with providing care to older parents—and secondary stressors, which result from the interaction of these tasks with other activities that may lead to conflicts between caregiving and other life responsibilities (Pearlin et al. 1997; Van den Berg et al. 2014).

Having a parent in a slow process of dying can also be a source of mental distress that negatively affects life satisfaction, as indicated by research on depressive symptoms (Amirkhanyan and Wolf 2006; Han 2023). Hence, the stress process model can include two sources of stress: those stemming from having a parent in need of care or in the process of dying, which can be classified as primary sources of stress (Zarit and Whitlatch 2023), and those stemming from providing care for older parents in these situations. Since the primary contribution of the current analysis focuses on the first set of processes (H1), we will delve more into this aspect, whereas the second (H2)—caregiving—has been described by several other studies (Pinquart and Sörensen 2004; Zarit and Whitlatch 2023).

Qualitative research shows that the physical and mental health of aging parents can negatively impact on their children’s emotional well-being and feelings of guilt as they witness the gradual decline in their parents’ functionality and independence (Luichies et al. 2021). While many adult children feel a deep sense of gratitude for the support and love provided by their parents throughout their lives, these feelings of gratitude and emotional bonds may also lead to emotional distress when faced with the reality of witnessing their parents’ health deteriorating (Labbas and Stanfors 2023). Despite the emotional and logistical challenges of providing support and care, most adult children express a sense of duty to support their parents during their final phase (Schulz and Eden 2016). This emotional complexity can be compounded by moral expectations and a personal desire to ensure the best care and support for their parents, even while knowing that their health will likely continue to decline.

Furthermore, the deterioration in health and eventual death of older parents often serves as a profound reminder of one’s own mortality and the frailty of life. Adult children may experience not only grief from the loss of their parents’ companionship and support but also an acute awareness of their own aging process and the prospect of future health deterioration and vulnerability (Umberson 2003). This realization can prompt existential reflections in which the sadness of losing a loved one is interwoven with an acknowledgment of old age, frailty, and life’s finiteness.

Both sources of primary stress—declining health in older parents, caregiving for older parents—may lead to secondary stressors, meaning that they affect other life domains such as employment, social life, and other family relations beyond older parents. The mechanisms linking the primary and secondary sources of stress are likely to vary; declining health and frailty in older parents are primarily associated with emotional stress that may impact other life domains, whereas caregiving affects these domains through the time and effort required by caregiving tasks.

Caregiving may not only have negative impacts on caregivers. For example, Folkman (1997) argued that the stressful situation of caring for a loved one who is in the process of dying is associated with both negative and positive emotions. This argument aligns with the development of positive psychology, which has identified several factors that can enhance well-being, including social ties with one’s family (Thomas et al. 2017). Strong family ties have been viewed as a major source of well-being by respondents (Delhey 2004), and providing support to family members is often positively associated with well-being (Thomas 2010). In fact, caring for older parents appears to be a notable exception to the more general finding that providing care is often associated with improved well-being (Pinquart and Sörensen 2006; Thomas 2010; Broese van Groenou et al. 2013). The reason for this exception may be that it is more stressful to witness a loved parent losing health and strength rather than providing care per se. For all these reasons, we consider H1—the emotional effect of observing a parent losing health and strength—a stronger hypothesis than H2—providing care to an older parent.

### Gender

Daughters tend to provide more care for older parents than sons, and this gender gap appears to persist across European countries and regions with different distributions of caregiving responsibilities between families and public care institutions (the availability of long-term care), as well as varying levels of female employment (Haberkern and Szydlik 2010:312; Labbas and Stanfors 2023:18). Women generally have greater life-course involvement with children and family than men (e.g., Anxo et al. 2011). These findings may indicate that social expectations for filial care of older parents are stronger for daughters than for sons. If so, it can be expected that the emotional stress stemming from poor health and the caregiving needs of older parents will be felt more acutely by daughters than by sons. For these reasons, we hypothesize (H3) that poor health and caring needs of older parents have less beneficial effects on the life satisfaction of daughters compared to sons.

### The changing role of family care

How frail parents affect the well-being of adult children may depend on institutional characteristics regarding who is responsible for providing care for older people. This has traditionally been the responsibility of the family and is often done by spouses and adult children. In affluent societies, including those in Northern Europe, care for older individuals has gradually been taken over by professional caregiving institutions (Brandt et al. 2009; Christensen and Wærness 2018). In Norway, the provision of long-term care for frail individuals is formally the responsibility of the municipalities, and filial obligations have been removed from legislation (Skinner and Sogstad 2022).

Public care institutions will typically not replace all types of support offered by family members. At the most basic level, frail older individuals may need personal care for daily activities such as dressing, bathing, and eating, often labeled as Activities of Daily Living (ADL). Frail older individuals may also need practical support for less frequent activities such as travel and shopping, preparing food, paying bills and other financial support, and cleaning and maintaining a home, labeled as Instrumental Activities of Daily Living (IADL) (Brandt 2013). Finally, this group may also need social support, and relationships with family members could become particularly important as they reach an age when they perceive their time as limited, as argued by socioemotional selectivity theory (Carstensen et al. 1999; Hashiguchi and Llena-Nozal 2020).

Comparative data show that relatively few people in Norway and other Nordic countries provide the most basic forms of personal care (Ambugo et al. 2021; Schönfelder et al. 2020; Tur-Sinai et al. 2020). On the other hand, practical help and social support for older parents are widespread in the Nordic countries, more so than in other European countries (Brandt et al. 2009; Motel-Klingebiel et al. 2005). These findings indicate that when governments take responsibility for providing care for older individuals, the moral expectation of providing filial care is relaxed primarily for more basic and mostly physical forms of care, i.e., for the so-called ADL functions and certain IADL functions. The family still has an obligation to provide other types of practical help (including for other IADL functions) and social support to its older members, which professional workers cannot prioritize (Brandt 2013; Daatland and Herlofson 2003; Kääriäinen et al. 2014).

## Method

Our empirical analysis investigated data from rounds 2 and 3 of the Norwegian Life Course, Ageing, and Generation Study (NorLAG) (Veenstra et al. 2021), collected in 2007/08 and in 2017/18. These two NorLAG rounds include harmonized assessments of factors we study, including life satisfaction and intergenerational relations. In both rounds, data were collected using initial telephone interviews followed by self-administered postal questionnaires. Response rates for the two telephone interviews were 61% and 68%, respectively, of which approximately 75% also completed the self-administered questionnaires (both rounds).

The outcome variable was life satisfaction, measured by four items from the Satisfaction with life scale (Pavot et al. 1991). These items were: “In most ways, my life is close to ideal;” “My living conditions are excellent;” “So far, I have gotten the most important thing I wanted in life.” The three items were included in the self-administered postal questionnaire, whereas the fourth item was from the initial telephone interview: “I am satisfied with my life.” Five response categories varied from “Strongly agree” to “Strongly disagree.” The satisfaction with life scale was standardized with a mean of 0 and a standard deviation of 1. The statistical analysis included all respondents with valid data on life satisfaction in both rounds of data collection (2007/8 and 2017/18), aged 40 (restricted by data source) to 77 years (no respondents above this age had parents alive).

The main explanatory variables were a set of indicators related to having a parent with various levels of health-related needs and providing care oneself. These indicators were collected during telephone interviews, starting with: “Is your mother/father (separate questions for mother and father) in any way limited in her/his daily activities due to ill health, mental health problems, or a disability?” If the answer was yes, respondents were asked, “Are these limitations of extensive degree, some degree, or minor degree?” Here, values were assigned from 0 (no limitations in either parent) to 3 (extensive limitations in at least one parent). Further questions included: “Is your mother/father in need of help for daily activities?” (such as housework, travel and shopping), and “Does your mother or father need personal care?” This was specified as “such as eating, standing up, dressing, or walking to the toilet.” Finally, if the answer was yes to either of the two previous questions, respondents were asked, “Who is helping your mother/father?” with several categories to choose from, including the respondent.

These questions led to the construction of three binary items: having a parent in need of help (15.7%), having a parent who needs personal care (9.1%), and the respondent giving such help or care (2.6% of the observations, as shown in Table 1). The latter item (giving care) was slightly more frequent among female (2.9%) than male respondents (2.2%), and for simplicity, it is labeled “gives care” in the following text.^1^

**Table 1:** Descriptive statistics of the data, both genders (6,188 observations)

	Mean	S.D	Low	High
Life satisfaction	0.0	1.0	− 4.5	1.5
Age (years)	58.2	9.0	40	77
Parents limitations	0.63	1.1	0	3
Parents need help	15.7%		0	1
Parents need care	9.1%		0	1
Participant gives care	2.6%		0	1
Employed	65.3%		0	1
Partnered	77.6%		0	1
No parent alive	53.2%		0	1

The data were analyzed longitudinally using linear regression models with fixed effects for individuals, providing ‘within-individual’ change estimates. This method accounts for all time-invariant or ‘fixed’ characteristics of the participants. Because changes in life satisfaction (and most other outcomes) tend to entail long tails—most people change little, and those who do change can alter significantly—all statistical tests were robust for heteroscedasticity.

All statistical models controlled for the respondent’s age through linear and squared terms, as well as the situations of no longer having any parent alive (53.2% of the observations), being employed and partnered (married or cohabiting). Initially, separate models were used to investigate each of the health-related factors of the participants’ parents and the respondent’s caregiving in separate models for sons and daughters (Table 3). Subsequently, the analysis was conducted jointly for the help or care needs of a parent and for caregiving provided by the respondent (Table 4). Interactions between gender and each of the parents’ health-related factors were estimated using interaction terms in pooled models for sons and daughters. (Table 5). A final analysis (Table 6) also distinguished between entering and exiting the situations of having parents in need of care and giving care oneself.

Because our longitudinal estimates were only affected by participants who took part in both the 2007/08 and 2017/18 waves, we report only the number of participants (a total of 3,094 individuals) in the tables with regression results; the number of observations is twice this number (6,188). Of these 3,094 individuals, 1,188 (38.4%) experienced a net change in their parents’ health limitations, 757 (24.5%) experienced a net change in their parents’ need for help, 507 (16.4%) experienced a net change in their parents’ need for care, and only 146 (4.7%) changed caregiving (Table 2). All these numbers were higher for exiting these situations than for entering them, primarily because one or both parents had died between the two rounds of data collection (Table 2).

**Table 2:** Descriptive statistics for changes and number of transitions between the two waves of data (3,094 individuals)

Continuous/discrete variables:	Mean change	S.D. change	Low	High
Life satisfaction	0.12	0.9	− 4.5	4.1
Age (years)	9.72	9.0	8	11
Parents limitations	− 0.30	1.4	− 3	3
Indicator variables:	Changed		#Entering	#Exiting
Parents need help	24.5%		298	459
Parents need care	16.4%		205	302
Participant gives care	4.7%		65	81

## Results

Having parents who needed help or care made the daughters, but not the sons, less satisfied with their lives (Table 3). Health-related problems in older parents affected their adult children less than having parents in need of help and care. Further, having parents who needed care appears to have affected their daughters’ lives more than merely having parents who needed help.

**Table 3:** Life satisfaction related to four measures of having parents in poor health or in need of help/care, from separate models (1–4) that controlled for age (linear and squared terms), being employed, partnered, and having no parent alive; longitudinal (fixed effects) estimates

		Men		Women	
Model	Parents …	Coeff	SE	Coeff	SE
1	.. limitations (0–3)	0.031	(0.021)	− 0.004	(0.022)
2	.. need help (1,0)	0.016	(0.055)	− 0.112*	(0.056)
3	.. need care (1,0)	0.012	(0.056)	− 0.185**	(0.062)
4	Participant gives care (1,0)	0.152	(0.118)	− 0.052	(0.121)
	Individuals	1,478		1,616	

* = *p* < 5%, ** = *p* < 1% (two-sided tests)

Providing personal care oneself did not seem to affect the life satisfaction of daughters beyond the negative effects of having a parent in need of help or care. This is probably better illustrated in Table 4, where these factors are estimated jointly, assuming that adult children provide help or care in situations where such support is needed. With sons, on the other hand, these results were different, and it was even possible that caregiving could be beneficial to the life satisfaction of sons (non-significant in all models in Tables 3 and 4).

**Table 4:** Life satisfaction related to having parents in need of help (Model 1) and care (Model 2) and giving care oneself (both models) with statistical controlled for age, employment, partnership status, and no parents alive; longitudinal (fixed effects) estimates

		Men		Women	
Model		Coeff	SE	Coeff	SE
1	Parents need help	− 0.002	(0.056)	− 0.115*	(0.058)
	Participant gives care	0.153	(0.121)	0.018	(0.125)
2	Parents need care	0.008	(0.056)	− 0.184**	(0.062)
	Participant gives care	0.151	(0.118)	− 0.026	(0.118)
	Individuals	1,478		1,616	

* = *p* < 5%, ** = *p* < 1% (two-sided tests)

The findings in Tables 3 and 4 indicate that men and women may be affected differently when an older parent needs care. While daughters are negatively affected in terms of life satisfaction, the average son is less affected. Only one of these comparisons between sons and daughters is statistically significant when tested using pooled models with interaction terms with gender (Table 5 in the Appendix), which arises in the situation when parents need care.

**Table 5:** Life satisfaction related to four measures of having parents in poor health or in need of help/care, from pooled models (1–4) with interaction effects with gender (female dummy) that controlled for age (linear and squared terms), longitudinal (fixed effects) estimates controlling for age, employment, partnered, and having nor parents alive; 3,094 individuals

		Men (main effect)		Women–men (interaction)	
Model	Parents …	Coeff	SE	Coeff	SE
1	.. limitations (0–3)	0.022	(0.019)	− 0.019	(0.022)
2	.. need help (1,0)	− 0.004	(0.051)	− 0.089	(0.064)
3	.. need care (1,0)	− 0.010	(0.054)	− 0.153*	(0.075)
4	Participant gives care (1,0)	0.148	(0.116)	− 0.198	(0.163)

* = *p* < 5% (two-sided tests)

It is also possible to distinguish between the processes of entering and leaving these situations (Table 6 in the Appendix). Our main finding of how daughters are negatively affected when older parents need care is seemingly driven by both entering and exiting effects as these are of similar magnitude. In sons, on the other hand, there seem to be more complicated processes as exiting caregiving can in certain situations, when parents are still alive, negatively affect their satisfaction with life.

**Table 6:** Full regression results of changes in life satisfaction corresponding to model 2 in Table 4 that also separates between entering and exiting situations where older parents need care and where participants give care

	Men		Women	
	Coeff	SE	Coeff	SE
Parents enter need of help	− 0.026	(0.090)	− 0.161	(0.088)
Parents exit need of help	− 0.045	(0.075)	0.202*	(0.088)
Participant enters caregiving	− 0.066	(0.220)	− 0.104	(0.170)
Participant exits caregiving	− 0.303*	(0.128)	− 0.048	(0.164)
Age (years/10)	0.087	(0.070)	0.068	(0.073)
Age squared	0.012	(0.017)	0.020	(0.019)
Employed	0.031	(0.051)	0.084	(0.050)
Partnered	0.242**	(0.084)	0.142	(0.083)
No parent alive	0.133*	(0.058)	0.017	(0.066)
Constant	− 0.502	(0.104)	− 0.326	(0.095)

* = *p* < 5%, ** = *p* < 1% (two-sided tests)

## Discussion

Previous research indicates that providing care for older parents is associated with lower levels of subjective well-being, as indicated by happiness or life satisfaction (Hajek and König 2016; Kalenkoski and Oumtrakool 2017; Van den Berg et al. 2014). The current study found significant changes in life satisfaction when parents need care, but only small changes when participants provide care for older parents in situations where care is needed. The small number of participants providing care makes it difficult to obtain precise estimates of how caregiving affects satisfaction in adult children. Still, our data suggest that it is not caregiving per se that affects life satisfaction, but rather the circumstances leading to caregiving. In these situations, it does not seem to matter much who provides care—whether the respondent or someone else, as this is not associated with the life satisfaction of the child.^2^

Most likely, factors such as decreased functionality and helplessness in parents, along with the realization that they are undergoing a process that will lead to their death, have contributed to the association observed between declining life satisfaction in adult daughters and the needs for help and care of older parents. Additionally, these factors may also have contributed to previous findings of decreasing life satisfaction among adult children, rather than caregiving itself. If so, the statistical associations between caregiving and subjective well-being in prior research may also be causally spurious, reflecting factors not observed or specified in those statistical analyses.

For policymakers and practitioners in health and social services, it is important to understand that the adult children of old and frail people may struggle with sources of distress beyond those related to supporting and caring for frail parents. This distress also includes the stress of witnessing loved ones lose their health and eventually their life. It may even prompt existential reflections and a realization of vulnerability and the finiteness of life.

Regarding research, there could be a lack of empirical sensitivity in research on how various situations and events may affect subjective well-being. There is a possibility that too much research has made overly strong theoretical assumptions regarding how situational factors affect various outcomes, rather than carefully inspecting these relationships in available data. This could have led researchers to construct social realities that may have appeared different if they had been more sensitive to the various situations and processes that might give rise to the outcomes investigated. One implication is that researchers should be more aware of alternative explanations for their main hypothesis, as argued by the well-known (but often overlooked) principle of falsification (Popper 1934 [1959]).

Our findings indicate that men and women may be affected differently when an older parent needs care. While daughters are negatively affected in terms of life satisfaction when older parents need care, the average son is less affected. This finding might suggest that the moral expectation of providing filial care has a gendered component, with more being expected of daughters than of sons.

Moral expectations related to filial responsibility could be felt as a burden by many women when their parents need care. Including Norway, there are stronger expectations that daughters will provide help and care than there are for sons (Costa‐Font et al. 2016; Jakobsson et al. 2016). The idea that the responsibility of filial care falls disproportionately on daughters is well known among policymakers. The expansion of public care was partly motivated by a preference for individual freedom and reducing the need for women to curtail or end their work careers when parents needed support (Haley and Elayoubi 2024; Jensen and Rathlev 2012). The empirical support for this social expectation explanation is limited; in the current study, it has not been tested directly in the data. Because the situation of having frail parents who need care appears to affect sons and daughters differently, it should be the subject of further empirical inquiry.

This study has investigated how the cognitive components of well-being change when older parents enter or leave situations of various levels of needs, as well as when adult children provide care. Future research could explore further aspects and indicators of well-being, such as positive and negative affect.

Our empirical analysis included a limited set of additional factors that can also change over the course of people’s lives (own age, employment and partnership status, no longer having any parent alive). Our main findings changed little when adding these covariates into the statistical analyses (not shown in the tables). This observation indicates that our main findings are robust about such modeling issues.

Our data do not provide information about the timing of the transitions investigated, and the time gap between the two observations for each participant is about 10 years. There is a possibility that the death of parents, or the process leading to their death, is associated with stress that differs from the stress of having parents who need care. Such temporal processes could have been examined with more precise timing of these transitions.

Our data were collected in a context of relatively generous public support for eldercare and high female employment rates. Future research should also investigate how far these situations affect sons and daughters in countries with different institutional characteristics and levels of female employment. There is a possibility that the increasing role of governments in providing basic care to older individuals in countries with extensive public care has led to the result that when adult children also provide some care to older parents, this caregiving burden is not sufficiently large to negatively affect their life satisfaction. Other research has shown that the gap in depression between caregivers and non-caregivers varies between countries (Wolf et al. 2015), and a longitudinal study of Danish and Swedish data indicates that a similar gap in quality of life (using a composite measure) is associated with the generosity of public eldercare (Van den Broek and Grundy 2020). Hence, there is a possibility that institutional care of acceptable quality has reduced the emotional toll on adult children by decreasing their caregiving burden, and that similar comparisons could look different in countries where the proportion of older people using public care is lower.

## Data Availability

No datasets were generated or analyzed during the current study.

## Appendix

See Tables 5 and 6.

## Footnotes

1 These questions regarding having a parent in poor health, needing help or care, or giving care were only asked of participants not sharing a household with a parent. Other research on these NorLAG data has used a stricter measure of giving regular care to older parents, but including both resident and non-resident parents (e.g., Vangen 2021). Co-residence with older parents is rare in Norway, however, and it dropped from 0.9% in round 2 to 0.3% in round 3 of NorLAG (from 2007 to 2017). Only 11 (0.3% of the) participants entered co-residence with a parent between rounds 2 and 3, and none of these participants reported giving regular care to their parent in round 3. If we use the stricter measure of giving regular care, we get similar results to those reported for caregiving to non-resident parents, but with larger standard errors because of fewer cases. Hence, we only report the results relating to care given to non-resident parents.

2 99,5% of parents in need of care in 2017 (this information was not available in 2007) received care by someone. Hence, if the respondent did not provide care, other people provided some care in these situations.
